# Anatomically informed multi-level fiber tractography for targeted virtual dissection

**DOI:** 10.1007/s10334-022-01033-3

**Published:** 2022-07-29

**Authors:** Andrey Zhylka, Alexander Leemans, Josien P. W. Pluim, Alberto De Luca

**Affiliations:** 1 Biomedical Engineering, Eindhoven University of Technology, Rondom 70, 5612 AP Eindhoven, The Netherlands; 2 Image Sciences Institute, University Medical Center Utrecht, Utrecht, The Netherlands; 3 Neurology Department, UMC Utrecht Brain Center, University Medical Center Utrecht, Utrecht, The Netherlands

**Keywords:** Diffusion MRI, Corticospinal tract, White matter

## Abstract

**Objectives:**

Diffusion-weighted MRI can assist preoperative planning by reconstructing the trajectory of eloquent fiber pathways, such as the corticospinal tract (CST). However, accurate reconstruction of the full extent of the CST remains challenging with existing tractography methods. We suggest a novel tractography algorithm exploiting unused fiber orientations to produce more complete and reliable results.

**Methods:**

Our novel approach, referred to as multi-level fiber tractography (MLFT), reconstructs fiber pathways by progressively considering previously unused fiber orientations at multiple levels of tract propagation. Anatomical priors are used to minimize the number of false-positive pathways. The MLFT method was evaluated on synthetic data and in vivo data by reconstructing the CST while compared to conventional tractography approaches.

**Results:**

The radial extent of MLFT reconstructions is comparable to that of probabilistic reconstruction: $p=0.21$ for the left and $p=0.53$ for the right hemisphere according to Wilcoxon test, while achieving significantly higher topography preservation compared to probabilistic tractography: $p<0.01$.

**Discussion:**

MLFT provides a novel way to reconstruct fiber pathways by adding the capability of including branching pathways in fiber tractography. Thanks to its robustness, feasible reconstruction extent and topography preservation, our approach may assist in clinical practice as well as in virtual dissection studies.

**Supplementary Information:**

The online version contains supplementary material available at 10.1007/s10334-022-01033-3.

## Introduction

Diffusion MRI fiber tractography provides an opportunity to estimate fiber orientations through the Brownian motion of water molecules. This imaging technique allows for exploring brain connectivity in vivo and non-invasively [1, 2] as well as performing virtual dissection [3–6], aiding pre-surgical planning [7] and serving as a reference during surgery [8]. In case of neurosurgery planning, the extent of resected tissue may need to be limited in order to limit function deficit, despite maximal tumor resection being one of the key factors for prolonged survival [9, 10]. Consequently, fiber bundle reconstructions need to have adequate extent to enable clinicians to estimate a safe resection margin. Despite its promising results, fiber tractography remains challenging, as the results of existing methods have been shown to perform satisfactory on either sensitivity or specificity, but not both [11–13].

For the purposes of surgery planning and virtual dissection, the sensitivity of tractography plays a key role, as the correct prediction of the extent of resection is essential to avoid functional impairment. The corticospinal tract (CST) is one of the bundles which neurosurgeons and neuroradiologists focus on during surgery planning to prevent motor function degradation [14]. However, the reconstruction of the corticospinal tracts and other pathways are often limited by intrinsic flaws of existing tractography algorithms, which by design makes it challenging to reconstruct branching configurations, leading to an increased false-negative rate [15].

Multiple approaches have been proposed to reconstruct the organization of fiber pathways from the diffusion signal, with the most common being the estimation of the fiber orientation distribution (FOD) with spherical deconvolution techniques [16–18]. Based on the way tractography methods use the information provided by the FOD, they can be categorized as either deterministic or probabilistic. Deterministic approaches follow either the dominant diffusion (or fiber) direction [19] or one of the main directions that is the least deviating from the orientation of a previous step [16, 20]. On the other hand, probabilistic approaches typically sample and propagate orientations based on the FOD in the voxel [21]. Probabilistic methods can potentially reconstruct branching-like configurations and have been shown able to reconstruct more true-positive pathways than deterministic methods, but also tend to have a higher false-positive rate [11] that complicates their application in pre-surgical settings. For instance, given that directions are sampled from orientation distribution, each step introduces a bias in relation to the peaks of the distribution. Consequently, during propagation, the bias may be accumulated to the extent that the reconstructed bundle does not follow known internal topographic organization [22–26] or accumulates the volume of plausibly looking pathways that will influence the safety margin estimation during tumor resection. In contrast, deterministic methods cannot reconstruct branching configurations and are prone to generating false-negative results, but their results are reproducible by definition and straightforward to interpret. Another approach that has the potential of resolving the tractography issues related to bundle extent is global tractography (GT). GT reconstructs all white-matter fiber bundles at once by optimizing an energy function based on the diffusion data. This group of approaches aims at resolving local fiber orientations by modeling pathways as a chain of connected segments and maintaining or changing the connectivity of the segments based on the underlying data. Despite the issue of being computationally expensive and suffering from fiber pathways that sometimes do not reach the cortex, GT can show improved performance in some cases [27].

As it was already briefly mentioned, certain fiber bundles, such as the optic radiation bundle and the CST, appear to have specific topographic organization [22–26] which assigns function duties to parts of these bundles. Maintaining such internal organization appears to be a challenge for probabilistic tractography unless it is specifically taken into account [22]. This creates potential issues in cases when functional data is used for the placement of either a seed region or simply a region of interest, for instance, when direct electric or transcranial magnetic stimulation is performed, further complicating the interpretation of the tractography results. In such cases streamline representation becomes more important given an additional constraint on sub-bundles visiting finer white-matter and cortical landmarks.

Incorporating anatomical prior knowledge in the tractography might offer a viable solution to improve the quality of the CST fiber tractography, given that anatomical landmarks are well defined for this tract [6, 28, 29]. For instance, the bundle-specific approach MAGNET [30] has been previously shown to enhance the reconstruction of the optical pathways by enforcing a specific direction for tract propagation using user-defined regions of interest (ROI). A similar guidance of the fiber tracking can be achieved also using transcranial magnetic stimulation to find the brain regions responsible for specific functionality for the purpose of filtering fiber bundles related to those regions [31].

Most anatomy-aware approaches attempt, thus, to either improve the streamline propagation or to enhance the FOD estimation. However, the aforementioned methods do not exploit all information available in the FOD. For one, the possibility of incorporating branching configurations with high angular deviations along fiber trajectories is not taken into account by most existing approaches. This problem has been first investigated by introducing the concept of pathway splitting [32], but the proposed framework may suffer from a high false-positive rate due to complications of the splitting procedure.

**Fig. 1:**
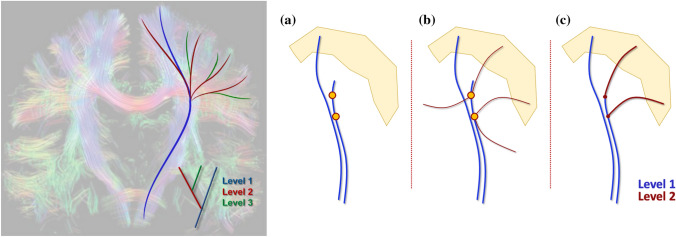
(Left) Current fiber tractography methods such as deterministic FOD-based tractography reconstruct only a subset of the pathways (blue). However, by propagating along the FOD orientations that were not used by a conventional tractography algorithm, the reconstruction can be iteratively extended by adding new sets of branches per iteration (red and green) leading to a final tractography result consisting of multiple levels. (Right) The pipeline of the algorithm. **a** The tract produced by the deterministic CSD-based tractography includes points with multiple FOD peaks, some of which are ignored. **b** Using these points as seeds with the unused peaks as initial locations, another iteration of CSD-based tracking is performed to obtain a new level of the result. **c** In the last stage only the tracts that enter the pre-defined target region are retained. The background picture on the left of the whole-brain fiber tractography result is taken from [33] with permission

In this work, we propose a novel approach to fiber tractography that adds branches to fiber pathways in a hierarchical multi-level approach (Fig. 1). By defining target and seed regions based on anatomical priors, the algorithm imposes additional constraints on the reconstructed streamlines, limiting the number of false-positive reconstructions that might be introduced either by the algorithm or via branching. Additionally, to differentiate crossing and branching configurations, only if a pathway does not reach the target, the peaks of the corresponding FODs may be considered as branches. This concept can be integrated into a wide range of tractography algorithms, e.g., any algorithm based on an FOD both probabilistic and deterministic. In this work, we focus on the proposed multi-level strategy in combination with deterministic constrained spherical deconvolution (CSD)-based tractography [20].

## Methods

### Multi-level fiber tractography

The core of our algorithm is a multi-level fiber tractography (MLFT) strategy that is compatible with a wide range of fiber tractography methods to take potential branching configurations into account. It is an iterative procedure that is capable of generating multiple spurious pathways and, consequently, requires user-defined starting and target regions as well as stopping criteria to control false-positive rate. MLFT can be combined with both deterministic and probabilistic methods. In name of clarity, in this first work, we choose to focus on combining our tractography strategy with deterministic CSD-based streamline tracking [20].

Our algorithm iteratively expands the reconstruction by branching from the pathways not reaching the target region. The set of streamlines visiting target region and added at one iteration can be considered one level of the overall bundle reconstruction. At each iteration, conventional deterministic CSD-based tractography is performed while storing information on which peaks were chosen for propagation at each point. If a reconstructed streamline does not enter the user-defined target region, its points that correspond to FODs with unused peaks are used as seeds for a new tractography level. Initial directions are defined as the FOD peaks that were not used during the reconstruction of the previous levels. The algorithm runs for a pre-defined number of levels or until a pre-defined convergence criterion is met. Finally, tracts that do not enter the target region at any of the considered levels are discarded (Fig. 1), which is a critical step to prevent the generation of aberrant branches. Co-existence of fiber crossings and fiber branching is facilitated by treating FOD peaks as crossings during propagation and only considering them as potential branches at the following iteration if a corresponding pathway does not reach a target region.

### Data

We performed experiments on both simulated and acquired diffusion weighted images. A numeric phantom was generated using *ExploreDTI* [34] (v4.8.6; PROVIDI Lab, Utrecht, the Netherlands; http://www.exploredti.com/) with 6 volumes at $b=0\, \mathrm{s}/{\mathrm{mm}}^{2}$ and 60 volumes at $b=1200\, \mathrm{s}/{\mathrm{mm}}^{2}$ with a resolution of 1 mm isotropic (Fig. S1 in Online Resource). The phantom represented three fiber bundles with two branching spots, conceptually mimicking fiber configurations as those that can be observed in the CST. The experiments with this phantom were performed without noise and for two signal-to-noise ratio (SNR) levels: 25 and 15.

To analyze the performance of our method on in vivo brain images, the *MASSIVE* [35] dataset was used. The data consisted of 430 volumes at $b=0\,\mathrm{ s}/{\mathrm{mm}}^{2}$, 250 volumes at $b=500\,\mathrm{ s}/{\mathrm{mm}}^{2}$, 500 volumes at $b=1000\, \mathrm{s}/{\mathrm{mm}}^{2},2000\, \mathrm{s}/{\mathrm{mm}}^{2}$ and $3000\, \mathrm{s}/{\mathrm{mm}}^{2}$ each, 600 volumes at $b=4000\,\mathrm{ s}/{\mathrm{mm}}^{2}$. The data were acquired with a resolution of 2.5 mm isotropic. The MASSIVE dataset was corrected for signal drift [36], subject head motion, eddy current and echo-planar imaging distortions [37].

Additionally, we applied our method to the preprocessed data of ten subjects from the Human Connectome Project (HCP). The data had a resolution of 1.25 mm isotropic and contained 18 volumes at $b=0\, \mathrm{s}/{\mathrm{mm}}^{2}$ and 90 volumes at $b=1000\, \mathrm{s}/{\mathrm{mm}}^{2}$, $2000\, \mathrm{s}/{\mathrm{mm}}^{2}$ and $3000\, \mathrm{s}/{\mathrm{mm}}^{2}$ each.

Multi-shell CSD [38] was used for the FOD estimation. The motor cortex was segmented as a combination of the left and right precentral and paracentral gyri (Fig. S2 in Online Resource) with *FreeSurfer* [39–41] (v6.0.0, Laboratory for Computational Neuroimaging, Charlestown, MA, USA; http://surfer.nmr.mgh.harvard.edu) and was used as a target region.

### Experiments

#### Experiment 1: Tractography in silico

We evaluated MLFT as well as iFOD2 [42], as it is a popular choice of probabilistic tractography algorithm, using a noiseless phantom. In all the experiments the implementation of iFOD2 from the MRtrix package [43] was used and all the options were set to default except for providing the seeding region. The same seed point was used for tracking in both cases.

The endpoint regions were placed at the separate ends of each sub-bundle (Fig. S1 in Online Resource) that served as target regions of interest for MLFT. They were also used to select the target fibers from the results of iFOD2, which was run with default parameters. The parameter setup for MLFT was as follows: angle threshold = 45°, maximum order of spherical harmonics ${L}_{max}=8$, FOD peak value threshold = 0.1, the default value in ExploreDTI. The step size was set to half the voxel size and the number of iterations was set to two.

#### Experiment 2: Robustness to noise

The sensitivity of the MLFT to noise was tested. Fiber tracking was performed for the phantoms at varying SNR levels with the same settings as in Experiment 1. The target fibers were then compared across SNR levels.

#### Experiment 3: Tractography in vivo

The MLFT approach was used to delineate the CST with the MASSIVE and HCP brain data described above. The motor cortex area of both hemispheres was used as a target region. The added value of our multi-level strategy was investigated more closely on the fanning projection of the left CST.

To evaluate whether MLFT reconstructs parts of the pathways belonging to the corpus callosum (CC), the bundle was delineated with both deterministic CSD-based whole-brain tractography and MLFT. The overlap of the CST and the CC generated by MLFT and CSD-based whole-brain tractography, respectively, was visually evaluated. The results of our algorithm were evaluated along with the results produced by the conventional deterministic CSD-based tractography from ExploreDTI as well as iFOD2 and GT [44] implemented in MRtrix. The CSD-based tractography, MLFT and iFOD2 used the same seed regions. The streamlines reconstructed by iFOD2 were further selected to include only the tracts that visit the target cortical area. In the case of GT, the masks of the seed and target regions were used to delineate the CST from the whole-brain tractography. To improve the visual interpretation of the results, implausible streamlines were removed using identical exclusion regions for all methods in case of the MASSIVE dataset.

Radial extents of the reconstructed bundles were calculated. To do that, the area covered by bundles’ endpoints in the cortex was calculated given that coronal projection of the motor cortex defines a 90º segment. Obtained radial extents were compared per hemisphere using paired Wilcoxon signed-rank test with significance level $\alpha =0.05$. Additionally, density distributions of the endpoints in the motor cortex were evaluated per subject for each algorithm.

The tractography parameters were set as in Experiment 1. Reconstructions with two iterations (for both MASSIVE and HCP) and three iterations (only for MASSIVE) were performed with MLFT. To obtain the whole-brain reconstruction with GT, the number of iterations was set to 10^9^, segment length = 1.5 mm, maximum spherical harmonics order ${L}_{\mathrm{max}}=8$. The default values were used for the remaining settings.

To reconstruct the CST with the MASSIVE dataset, the seed regions were placed close to the internal capsule. In case of HCP subjects an axial cross section of the brain stem was used. For seeding 100 points per voxel were evenly distributed at a single slice level. The number of seed points per voxel was selected empirically.

To run iFOD2, the FODs that were used for MLFT and CSD-based reconstructions were converted to MRtrix format using MRIToolKit (Image Sciences Institute, UMC Utrecht, the Netherlands; https://github.com/delucaal/MRIToolKit). iFOD2 was provided with a mask of the seed region used for MLFT, *seed_image* option was used. When performing iFOD2 on the HCP data, the number of selected pathways was empirically set to 10,000. In addition, the target regions were provided using *include* option, while the same function was used for filtering as in MLFT in case of the MASSIVE dataset. During the analysis of the HCP data, a NOT gate was used to remove inter-hemispheric connections, due to the use of the common seed region in the brain stem for both of the CST branches.

#### Experiment 4: Topographic organization

Previous research has established that both the motor cortex and the internal capsule can be divided into regions corresponding to specific motor functions, and that such organization is preserved within the CST [25, 45]. The topography preservation index (TPI) [46] was calculated, which highlights whether pathways that pass in close proximity to each other through the internal capsule also have closely located endpoints in the motor area. This index reflects how well the internal organization is preserved in the bundle reconstruction. The lower the TPI score the more topographic organization is preserved in the reconstruction.

To calculate TPI scores, rectangular ROIs were defined around the left and right internal capsules, then the longest axis of the ROI was used to map all the tract points crossing the ROI onto [0; 1] segment. Consequently, each pathway is assigned a value ${v}_{i}\in \left[0;1\right]$, where $i$ is an index of a pathway. Afterwards, a triangulation is built using the endpoints in the motor area and each edge connecting two endpoints of pathways $j$ and $k$ is assigned a weight equal to the distance of the projections in the ROI: $w=\left|{v}_{j}-{v}_{k}\right|$. Finally, the TPI score is an average of the weights. The edge in the calculated triangulation signals close proximity of the endpoints in the motor area, while the weight serves as a penalty if the corresponding pathways' locations in the internal capsule are distant.

The TPI was computed for the left and right CST branches reconstructed by each of the algorithms. To visually appreciate such organization, the CST streamlines were colored according to the part of the area of the motor cortex they reach. This allows to visually check whether the pathways reconstructed by MLFT and iFOD2 on the MASSIVE data corresponded to the anatomical position of the same associated function in the internal capsule. Additionally, statistical testing was performed to compare obtained TPI scores using paired sign-rank Wilcoxon test with significance level $\alpha =0.05$.

#### Experiment 5: Anatomical plausibility

As the previous experiment evaluates topography preservation capability of the algorithms by comparing relative placement of the endpoints, the coherence of the pathways was evaluated in order to observe whether the geometric similarity between pathways closely located to each other along their length is associated with the calculated TPI scores. We hypothesize that a fiber reconstruction with a lower intrinsic geometric similarity corresponds to a higher TPI, highlighting the effect of the bias on fiber pathway propagation. To this end, the minimum average direct-flip (MADF) distance was employed, which previously has been used in bundle clustering applications [47, 48]. This metric represents the average point-to-point distance between two pathways and is invariant to the ordering of the points in each pathway (e.g., to which endpoint is considered the start/end). It is defined in the following way: ${D}_{AB}=\mathrm{min}\left(\frac{1}{N}{\sum }_{i=1}^{N}\left|\left|{a}_{i}-{b}_{i}\right|\right|,\frac{1}{N}{\sum }_{i=1}^{N}\left|\left|{a}_{i}-{b}_{N-i+1}\right|\right|\right)$, where ${a}_{i}$ and ${b}_{i}$ are the points of the pathways $A$ and $B$ of length $N$, respectively. The metric requires the compared tracts to contain an equal number of points, which is why all the pathways were uniformly resampled to $N=200$ points. Evaluations were performed on the left and right CST bundles of the MASSIVE and HCP data obtained by the tested methods without filtering gates. For each set of the reconstructed pathways of a given subject, an all-to-all distance matrix was calculated. Then, for each pathway, the minimum distance was calculated based on that matrix.

## Results

### Experiment 1: Tractography in silico

**Fig. 2:**
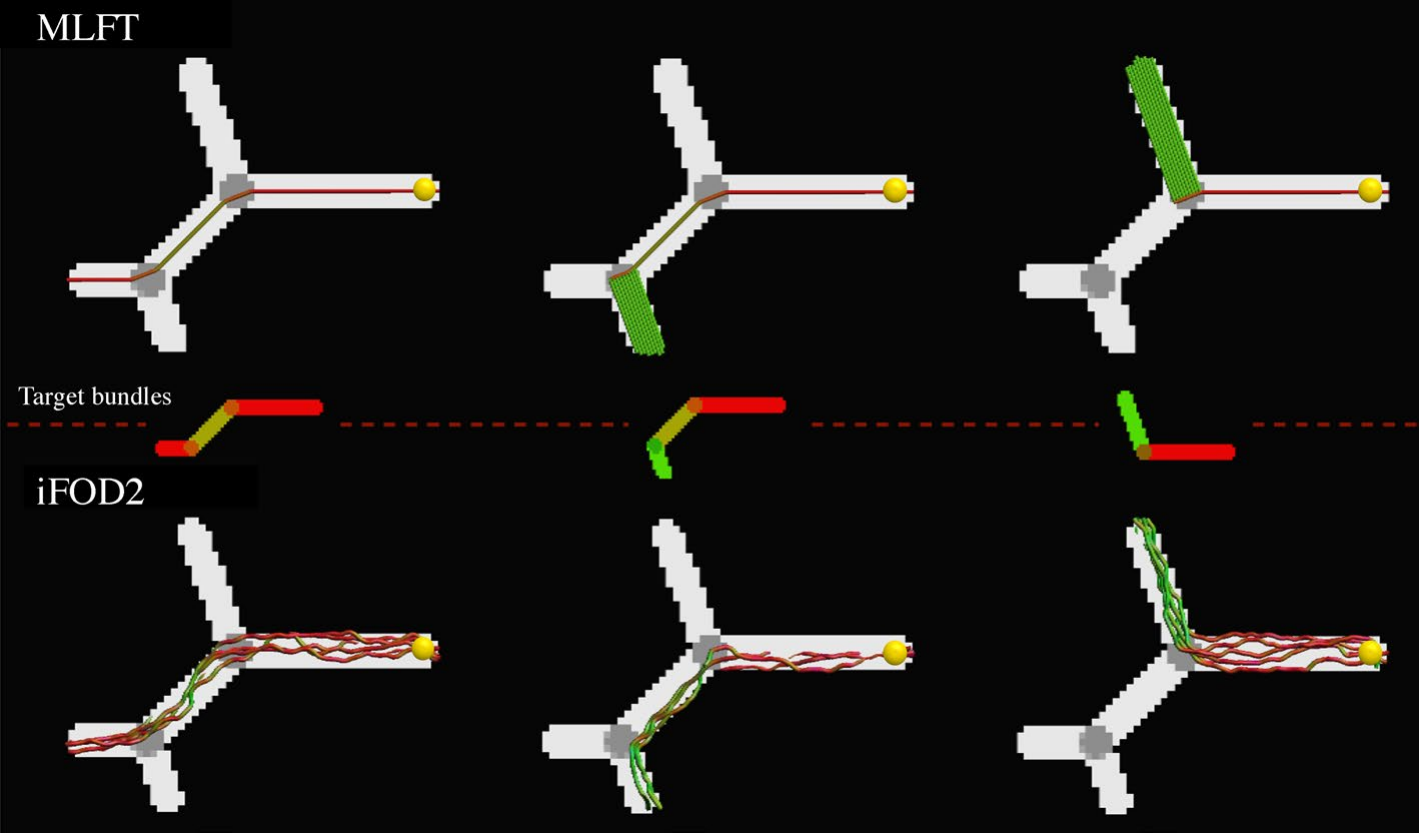
Performance of the considered methods in phantoms (FA map). The top row shows the results of MLFT and the bottom row those of the iFOD2 algorithm. The middle row illustrates the target fibers per column (orientation-based colored FA map). The same single seed point (yellow sphere) was used for both algorithms. The results of iFOD2 were subsampled for easier visual assessment. Streamlines' colors are based on orientation color-coding

Both MLFT and iFOD2 reconstructed all the phantom branches of the noiseless DWI phantom, as shown in Fig. 2. It can be observed that the results of MLFT follow the underlying simulated directions, whereas iFOD2 produces trajectories oscillating around the ground truth.

### Experiment 2: Robustness to noise

**Fig. 3:**
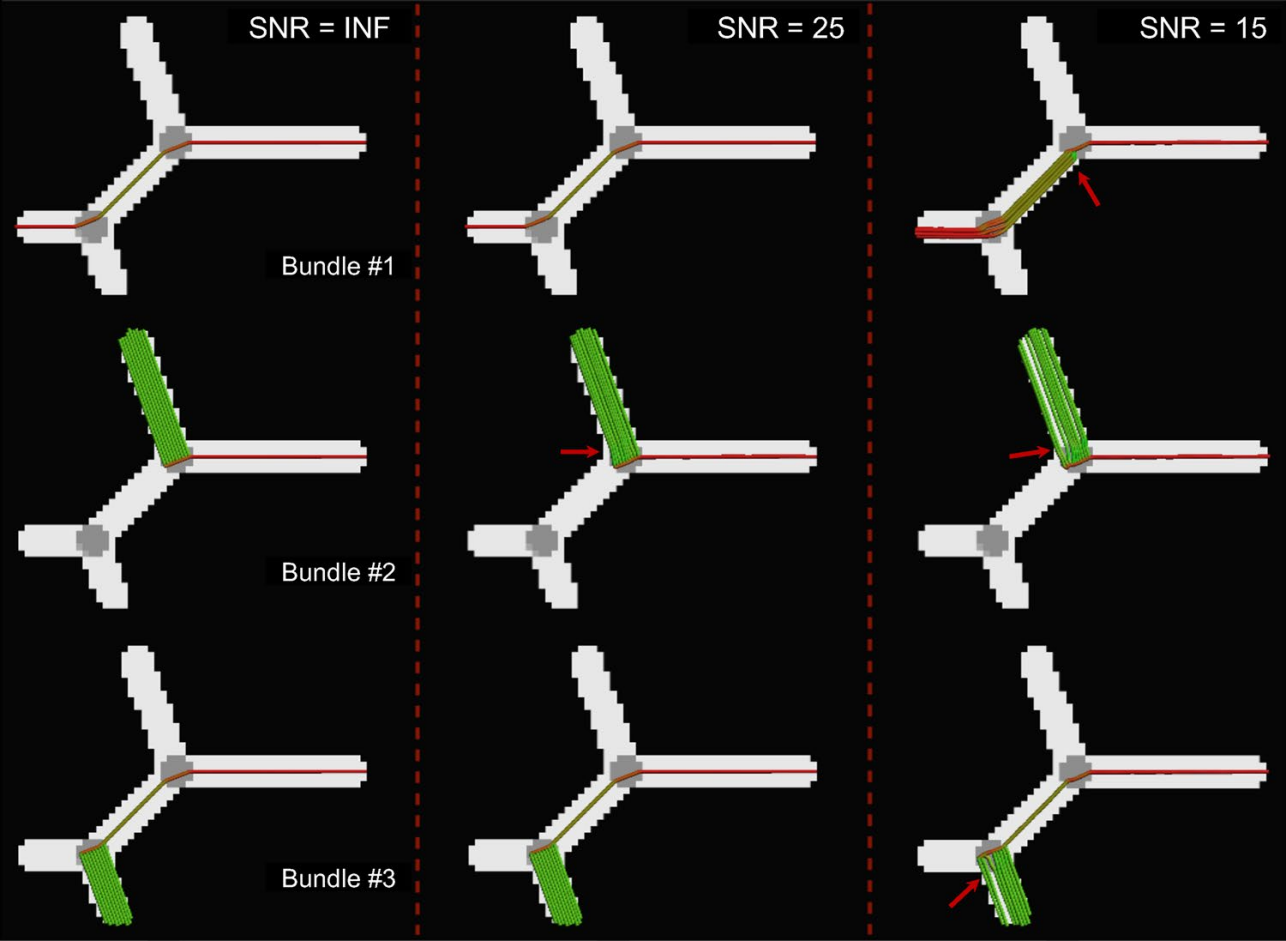
Tracts reconstructed by MLFT on the phantom data (FA map) at multiple SNR levels. Considerable angular errors are only observed at SNR = 15: increased number of branching configurations and direction perturbations up to 30º (red arrows). At SNR = 25, there is a minor angular deviation below 10º (red arrow). Streamlines are colored using standard orientation color-coding

The results of MLFT obtained for three different SNRs are presented in Fig. 3. A slight misalignment lower than 10° can be observed at the branching point at SNR = 25, which becomes more evident at SNR = 15 with values up to 30°. The case with the lowest SNR is also characterized by an increased pathway number of branching configurations at the points where original bundles diverge, as can be seen in the top row in Fig. 3.

### Experiment 3: Tractography in vivo

**Fig. 4:**
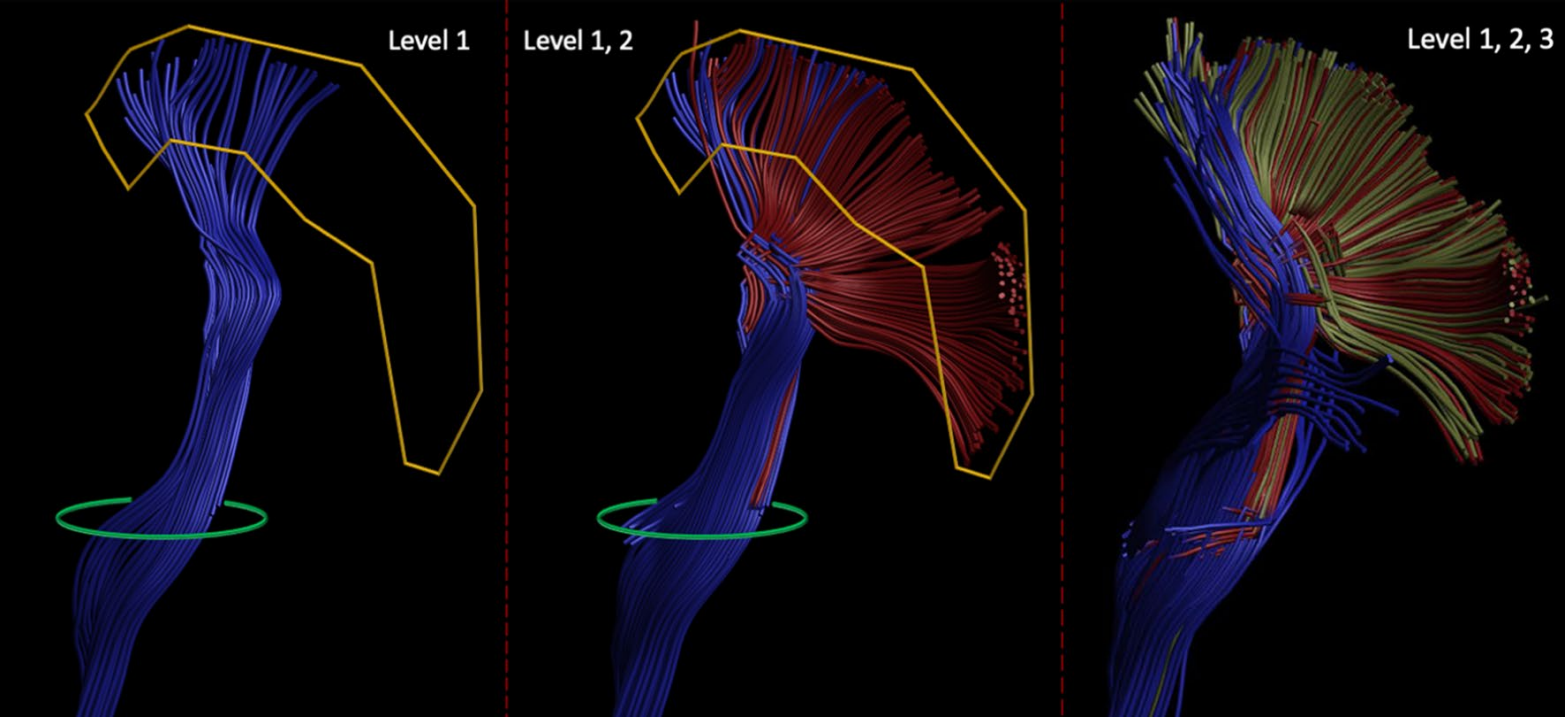
Fiber pathways reconstructed by the deterministic CSD-based approach (left) and MLFT with two (middle) and three (right) levels from the same seed region (green) with the same target region (yellow, the motor cortex) using MASSIVE dataset. Adding the second-level branches (red) to the pathways obtained at the first level (blue) improves the extent of the reconstructed bundle. Using three-level reconstruction from the same seed region does not show coverage improvements over the two-level reconstruction

The multi-level structure of the reconstructed left CST bundle can be seen in Fig. 4, which clearly shows the benefit of the proposed algorithm over conventional deterministic CSD-based tractography with the improved extent of the bundle fanning. The addition of an extra layer increases the number of streamlines reaching the motor cortex but does not bring further improvement to the coverage of the motor cortex: the radial extent with 3 levels amounts to 75.66º, while 2-level reconstruction has an extent of 71.48º. Consequently, in all of the in vivo experiments, the number of levels was set to two.

**Fig. 5:**
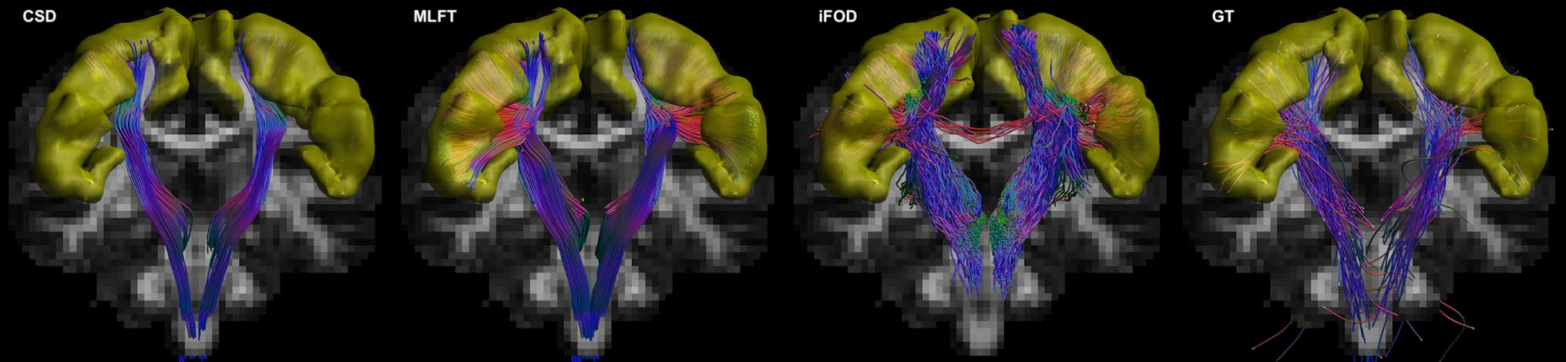
Corticospinal pathways reconstructed by the conventional deterministic CSD-based tractography, MLFT, iFOD2 and global tractography using the MASSIVE data. The motor cortex is shown in yellow. Some of the pathways reconstructed by iFOD2 enter the motor cortex and diverge into the CC propagating into the contralateral hemisphere

The full reconstructions of the CST segmented by MLFT, iFOD2, GT and deterministic CSD-based tractography in the MASSIVE data are shown in Fig. 5. It can be observed that the pathways obtained with MLFT densely cover most of the motor cortex unlike the results of deterministic CSD-based tractography. At the same time, both MLFT and iFOD2 cover most of the motor area (Fig. 5). For the iFOD2 reconstruction, the pathways traversing into contralateral hemisphere are present due to them bending after visiting the target region, returning into the white matter and propagating through the CC.

Regarding the reconstruction achieved by GT using the MASSIVE dataset, although the CST fanning is quite sparse, it reaches most parts of the motor cortex (Fig. 5**)**. The sparsity allows for a closer comparison of the multi-level and global tractography results which can be seen in Fig. S3. Unlike in the case of GT, the CST reconstructed by MLFT does not reach the approximate leg-related motor area. In the face area, the pathways generated by GT are aligned to those generated by MLFT, although they do not show any branching, but rather a smooth curving trajectory.

**Fig. 6:**
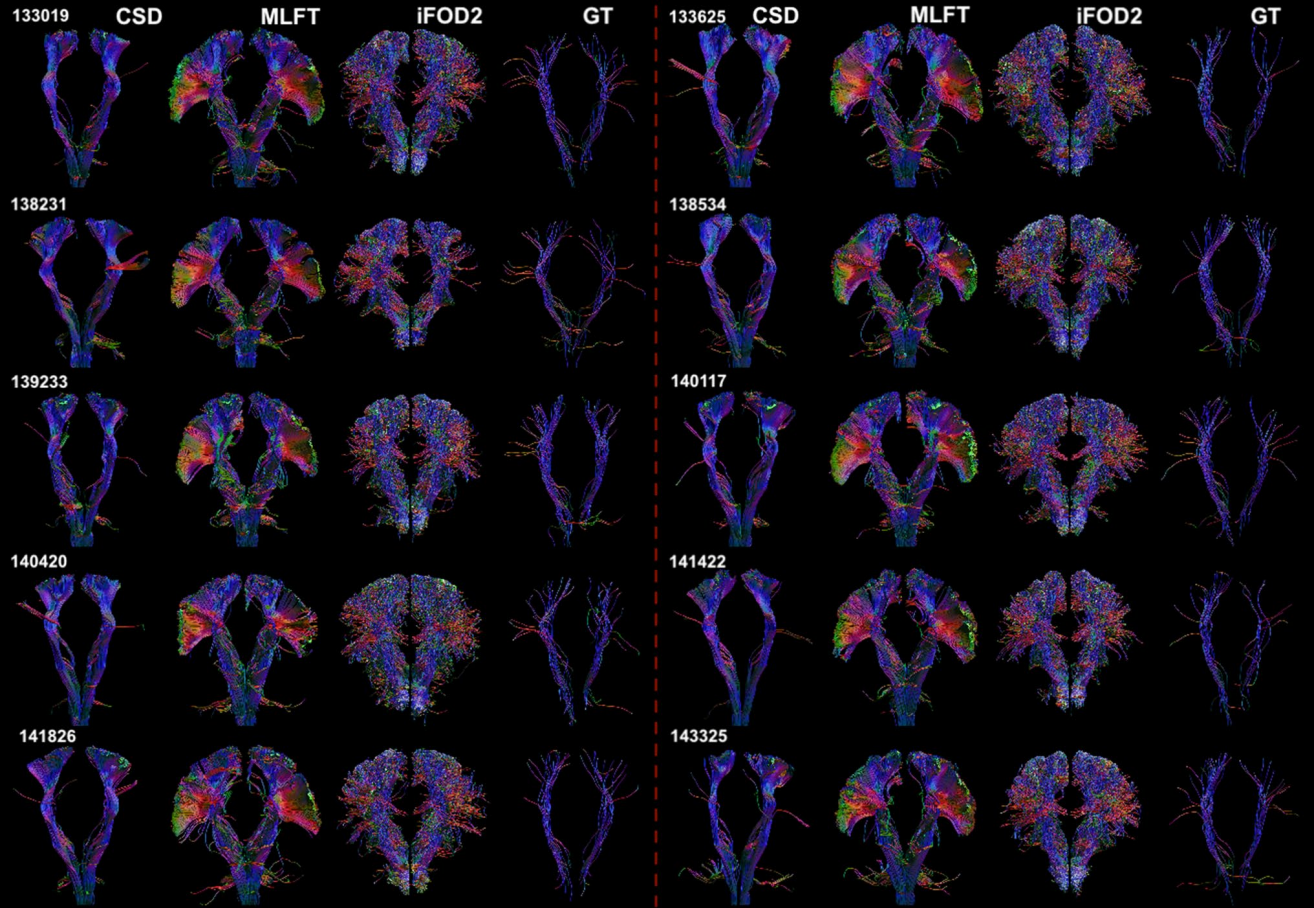
The CST reconstructions obtained by MLFT, iFOD2, GT and CSD-based tractography using the HCP data. The reconstructions by MLFT bundles are in line with the observations in Fig. 5 and consistent with each other. iFOD2 also achieves high motor cortex coverage. The extents of the GT-reconstructed bundles are comparable to the ones obtained by MLFT, but with less satisfactorily spatial coverage

**Fig. 7:**
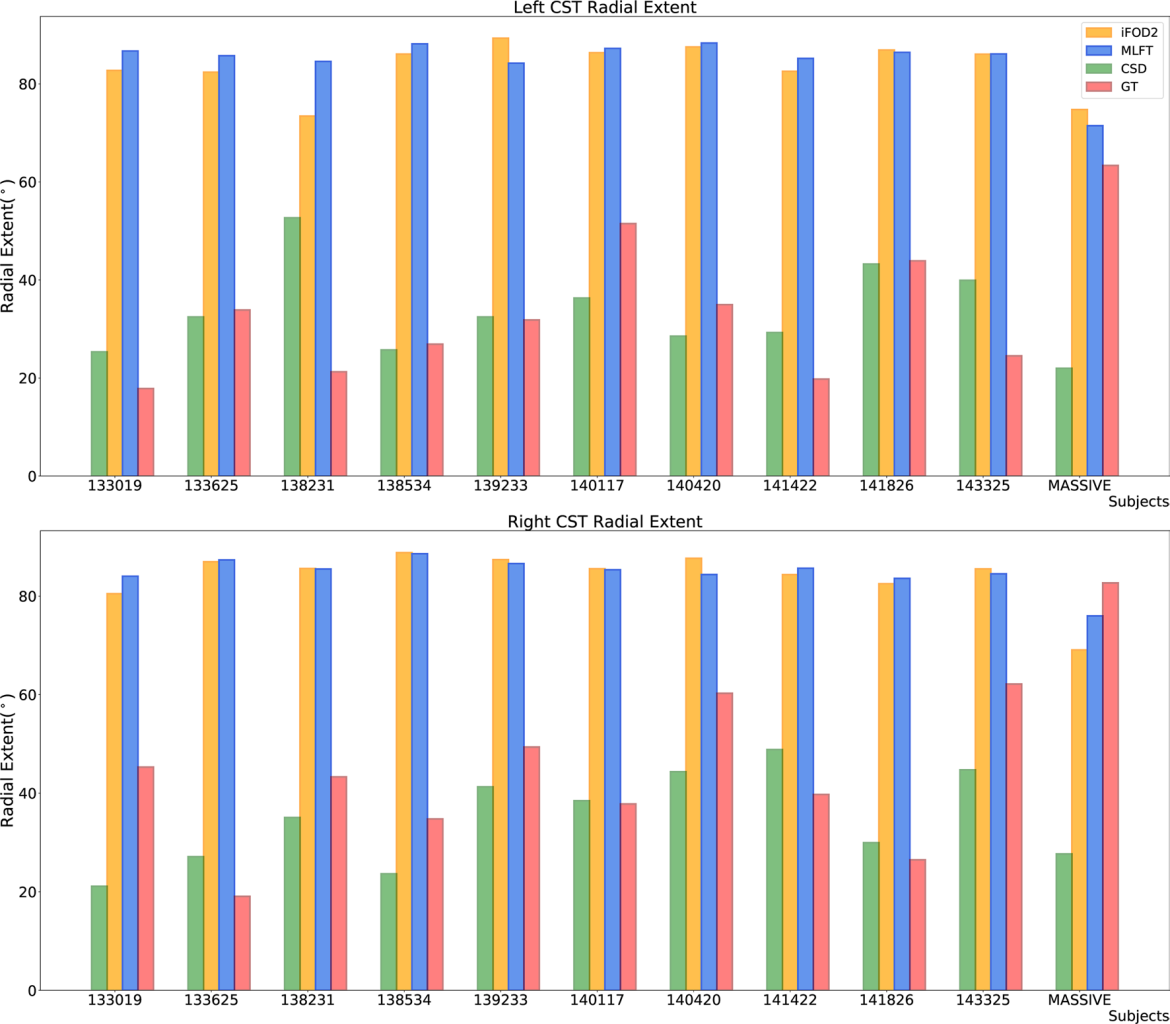
Radial extents of the reconstructed CST bundles for both left and right hemispheres. MLFT (blue) is shown to improve the radial extent compared to the conventional deterministic CSD-based tractography (green). iFOD2 (orange) and MLFT (blue) appear to have comparable radial extents. GT (red) achieves high radial extent on the MASSIVE dataset, while on HCP data the extent is primarily low

The CST bundles that were reconstructed for the HCP subjects by the proposed approach, iFOD2, GT and deterministic CSD-based tractography are shown in Fig. 6. Overview of the radial extents achieved by all the employed algorithms can be seen in Fig. 7. Regarding iFOD2, the results have the same characteristics as the results obtained using the MASSIVE data described above. Generally, both MLFT and iFOD2 reconstructions are represented by the bundles with a plausible fanning extent. GT seems to show lower radial extent compared to its result using the MASSIVE data.

**Fig. 8:**
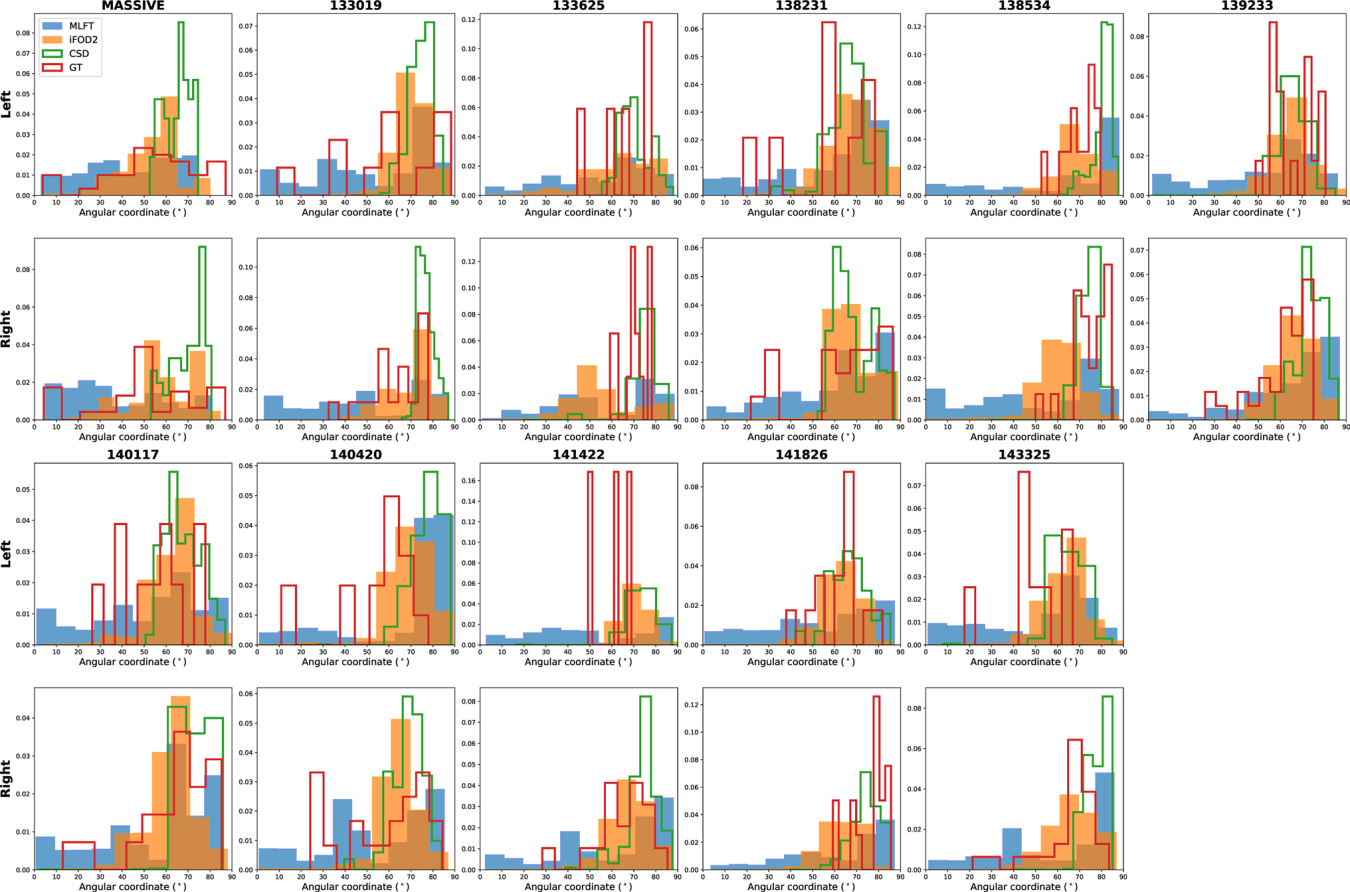
Density of the motor cortex coverage by the reconstructed CST bundles considering with angular coordinate starting at tempo-lateral point of coronal projection of the motor cortex and increasing towards superior motor cortex separately for each hemisphere. All the algorithms appear to densely cover superior part of the motor cortex. However, MLFT (blue) consistently covers most lateral part of the motor cortex with its density more evenly distributed compared to iFOD2 (orange). GT (red) also occasionally covers temporo-lateral motor cortex, although the coverage is very sparse. CSD-based tractography (green) primarily covers superior motor cortex part in all the subjects

Despite iFOD2 and MLFT both showing high radial extent, in case of iFOD2 the temporo-lateral part of the motor cortex is covered more sparsely than its superior part (Fig. 8**)**, At the same time, MLFT provides more uniform coverage of the motor cortex, although the superior motor cortex coverage is still relatively denser. Given the sparse reconstruction achieved by GT, its density distribution also appears quite uneven as can be seen in Fig. 8.

Performing statistical testing to compare the radial extents of the algorithms has shown no statistically significant difference between MLFT and iFOD2: $p=0.21$ for the left and $p=0.53$ for the right hemisphere. Also, no significant difference is observed between GT and CSD-based tractography: $p=1$ for the left and $p=0.06$ for the right hemisphere. All the other comparison combinations when performing Wilcoxon test resulted in $p$ values lower than the significance level.

The CST and CC bundles reconstructed using a subject from HCP data are depicted in Fig. S4 for comparison. In the axial view, it is well visible that part of the CST fanning does not overlap with the CC pathways, as the CC is not covering lateral part of the motor cortex.

### Experiment 4: Topographic organization

The TPI scores are reported in Table 1. The deterministic CSD-based tractography seems to outperform other algorithms showing lower values of the TPI metric, and thus higher coherence, for every subject. Both MLFT and CSD-based tractography achieved TPI scores that are significantly different from the scores of both iFOD2 and GT with $p< 0.001$. Despite rather close mean scores (0.03 and 0.06 for the left and 0.03 and 0.05 for the right CSD-based and MLFT reconstructions, respectively) MLFT and CSD-based reconstructions were shown to achieve significantly different TPI scores ($p< 0.001$). MLFT is shown to have seemingly comparable TPI scores to the CSD-based tractography, while they are still consistently lower than the scores of iFOD2 and GT reconstructions. In this regard, iFOD2 and GT show generally comparable performance to each other without statistically significant difference: $p =0.17$ for the left and $p =0.21$ for the right hemisphere.

**Table 1:** TPI scores of the left and right CST reconstructions by MLFT, iFOD2 and GT and also the TPI score of the first level of MLFT only, which is reconstructed by deterministic CSD-based tractography (the lowest score is indicated in bold)

Subject	Left				Right			
	CSD	MLFT	iFOD2	Global	CSD	MLFT	iFOD2	Global
MASSIVE	**0.08**	**0.08**	0.15	0.18	**0.08**	**0.08**	0.21	0.17
133,019	**0.03**	0.06	0.13	0.36	**0.02**	0.05	0.1	0.21
133,625	**0.03**	0.05	0.36	0.11	**0.03**	0.05	0.1	0.08
138,231	**0.02**	0.06	0.36	0.2	**0.02**	0.04	0.09	0.25
138,534	**0.03**	0.05	0.3	0.1	**0.03**	0.05	0.16	0.23
139,233	**0.02**	0.07	0.43	0.12	**0.03**	0.07	0.15	0.1
140,117	**0.02**	0.05	0.11	0.13	**0.02**	0.04	0.1	0.11
140,420	**0.04**	0.07	0.15	0.14	**0.03**	0.06	0.1	0.15
141,422	**0.03**	0.06	0.46	0.09	**0.02**	0.04	0.09	0.26
141,826	**0.02**	0.05	0.18	0.12	**0.02**	0.05	0.09	0.1
143,325	**0.02**	0.04	0.08	0.13	**0.02**	0.04	0.1	0.09

According to the TPI values, the CSD-based reconstruction of both CST branches has best-preserved topography. The scores of MLFT and CSD are comparable and consistently low in contrast to iFOD2 and GT

*TPI* topography preservation index, *CST* corticospinal tract, *MLFT* multi-level fiber tractography, *GT* global tractography, *CSD* constrained spherical deconvolution

**Fig. 9:**
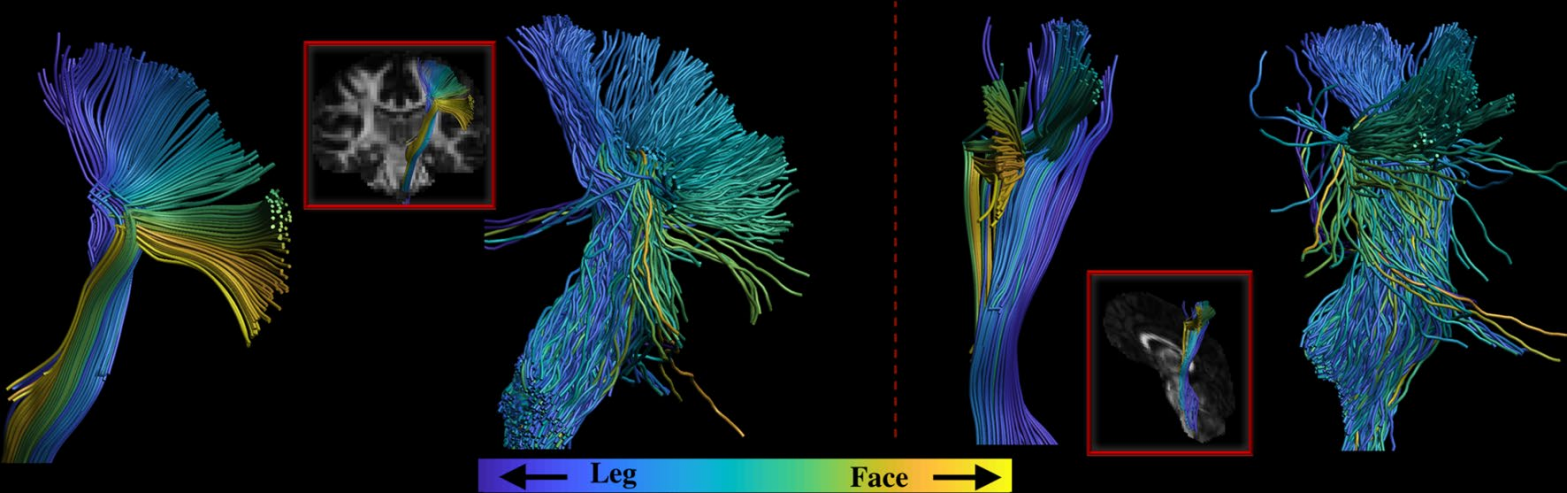
Coronal and sagittal views of the left CST reconstructed by MLFT and iFOD2 using the MASSIVE data. The fiber pathways are colored according to the locations of their endpoints in the motor cortex. The pathways reconstructed by MLFT are shown to have a clearer topographic organization

Figure 9 shows the pathways color-coded according to their final locations in the motor cortex. The visualization demonstrates that MLFT maintains the anatomical configuration of the pathways, according to which the organization of tracts connecting specific sub-domains of the motor cortex is maintained throughout the bundle. In contrast, the bundle produced by iFOD2 seems to be less organized.

### Experiment 5: Anatomical plausibility

**Fig. 10:**
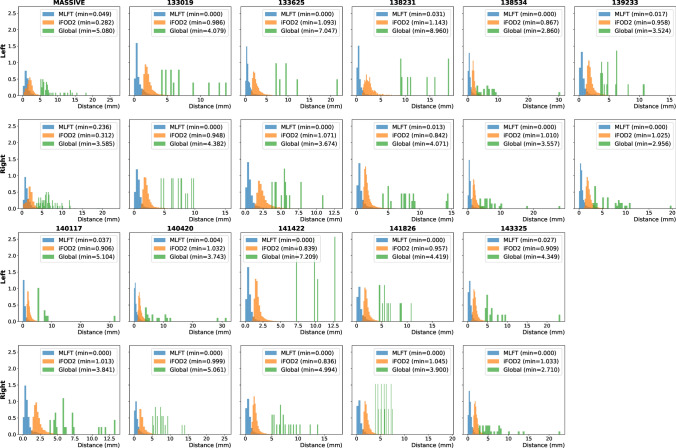
Normalized distributions of the distances from each pathway to the nearest neighbor based on the MADF distance for all the processed subjects. The distributions of the distances appear to be similar across subjects

The normalized histograms of the MADF distance are shown in Fig. 10. The distributions are similar across subjects per tractography approach and show that the distance between the closest pathways obtained by MLFT is generally smaller than that of iFOD2. The results of GT showed the highest distance, which is attributed to the sparsity of the bundles. The CSD-based reconstructions also appear to be very similar geometrically according to the MADF distance with the peak of the distribution being very close to zero for most of the subjects.

## Discussion and conclusion

In this study, we presented MLFT, a novel strategy to enhance fiber tractography by reconstructing branching configurations. The strategy we propose achieves anatomically plausible reconstructions of the CST bundles, is robust and reproducible, and maintains topographic organization. Each iteration of the proposed tractography algorithm attempts to branch existing streamlines towards the target region, which may open up new avenues for investigating more complex pathway configurations in the brain [49]. Given that image resolution is usually not sufficient to distinguish branching points, some of the FOD peaks might not only be an indication of crossing fibers, but also of branching ones.

Given the improved extent of the bundles and anatomically imposed control over false positives, our approach is attractive for a number of applications. It can be used to support pre-surgical planning, as it reveals more extensive coverage of the motor cortex than the conventional deterministic CSD-based tractography [50, 51], while maintaining clear structure of the reconstructed bundles.

### MLFT features

With simulations, we have shown that MLFT can reliably reconstruct branching fiber configurations that are less tortuous as compared to a probabilistic algorithm (Fig. 2). Additionally, the results of MLFT are reproducible. Although higher tortuosity of the probabilistic tractography reconstruction is an expected behavior, overcrowded reconstruction makes it a bit more challenging to spot spurious pathways. This also can be connected to the ability of the tractography algorithms to maintain topographic organization, which is relevant in applications involving brain stimulation methods, such as transcranial magnetic stimulation or direct electric stimulation.

Robustness to noise is another important aspect to consider. In order to analyze the sensitivity to noise of our algorithm, the same phantom bundles were simulated with three different SNR levels. The effect of SNR on the reconstructed pathways became clearly visible only at the lowest SNR level (SNR = 15), as reflected by an increased number of branching configurations and occasional perturbations after branching (Fig. 3).

When using the concept of branching for in vivo brain tractography, a well-delineated fanning was observed close to the motor cortex (Fig. 4). Although, MLFT and iFOD2 achieved comparable reconstructions (Fig. 7) of the CST fanning without statistically significant difference, iFOD2 reconstruction contains multiple spurious tracts (Figs. 5, 6). Apart from that, MLFT reconstructions show more uniform coverage of the lateral part of the motor cortex, while iFOD2 appears to provide much denser coverage of the superior motor cortex while sparsely lateral part (Fig. 8).

Most of the fanning consists of second-level branches, which might often look as if they diverge into another bundle at the branching points making a sharp turn. However, high angular deviations have been observed by Van Wedeen et al. [52]. Similarly, Mortazavi, et al. [15] also observed axon T-branching as well as sharp turns at sub-millimeter scale performing tract tracing experiments in the area under the motor cortex. Both of those papers present results based on the analysis of the macaque brain, but the statements are likely also valid for the human brain, which is reportedly congruent to the structure of the macaque brain [15], although it is difficult to provide estimates on the distribution of this type of branching in the human CST dissections. Additionally, certain cases can be considered a branching from a modeling point of view given the resolution. For instance, in case of the CST the fibers originating in the cortex descend into the trunk of the bundle. At this point, they pass through a "bottleneck" (at sulcus circularis insulae) and merge together [53]. As was presented in [54], up to 7 bundles appear to co-exist in a single voxel in the mentioned area, which would also suggest that some of the peaks may indicate a splitting of a bundle or an overlap of two bundles. Additionally, the angle between the CST trunk and the fanning close to the “bottleneck” area appears to be around 90° (Fig. 5 in [53]), which is usually absent due to the angular deviation threshold as propagation is incapable of making sharp turns. For this reason, probabilistic approaches struggle with reconstructing the inferior lateral part of the CST without smoothing the angle between the trunk and the fanning of the CST (Fig. 8). However, setting a threshold as high as nearly 90° for probabilistic tractography would overflow the result with false positives by allowing sampling not only around the FOD extremums.

The validity of the MLFT reconstructions can also be evaluated with Fig. S4. It is known that part of the CC originates from the motor cortex [29, 55] Thus, a successful reconstruction of the motor part of the CC remains prone to ambiguity as the CST pathways are present in that area as well. Similarity of the shapes of the MLFT-reconstructed bundles to those presented by Wasserthal et al. [56] provides additional confidence in plausibility of the results obtained by MLFT. Additionally, the comparison to the results of GT (Fig. S3) has shown that this alternative approach reconstructs similar pathways, although with certain smoothing of the high angular bifurcations that are observed in MLFT results. In general, the resemblance between the second level of the CST and the CC bundle can be explained by the co-alignment of the pathways of different bundles near the motor region reported by W. Krieg for the macaque brain and for the human brain [57]. This does not necessarily demonstrate that these similar pathways are true positive but serves as a reference which shows stable delineation of certain structures across various algorithms.

Specific topographic organization is a characteristic of a number of brain fiber bundles [23]. Somatotopic organization of the CST [24, 45] is one of the established examples of known internal bundle organization. Similarly to [22], we have evaluated the ability of the algorithms to maintain topographic organization using TPI score. According to the observed results, MLFT can preserve topographic organization of the fiber bundles as can be seen in Fig. 9. This is also reflected by the TPI scores (Table 1) across all the subjects analyzed in this study. The fact that topography preservation of MLFT is hampered compared to the deterministic CSD-based tractography might be a consequence of either obvious false-positive streamlines or precision mistakes, as in some cases first-level pathways terminate close to the target region and then branch at acute angles to reach it, and thus change the expected point location. By following the FOD peaks, we propagate the streamlines along the most reliable fiber orientation and, consequently, we are less affected by the noise. This leads to a more stable pathway propagation and, consequently, to a more anatomically reliable organization of the bundle. This is also supported by the presented higher values of pathway coherence of CSD-based tractography and MLFT compared to iFOD2 and GT (Fig. 10).

### Limitations

Some degree of uncertainty propagates in the results from the CSD procedure, as the response function is not voxel-wise perfect and FOD peaks have limited angular resolution. This limitation is, however, inherent to most tractography algorithms. Further, branching along a pathway might generate false-positive reconstructions. In our current implementation, correctly chosen anatomical priors are key to control the rate of false-positive pathways.

As revealed by the experiments with the phantom (Fig. 2), deterministic reconstruction from a single point may generate a whole dense branch, leading to an unrealistic density distribution. This is a result of the current implementation choice, of not imposing an upper limit to the number of times a streamline is allowed to branch. We believe that this aspect could be potentially improved in future work, for example, with a microstructure-informed extension of our framework.

### Methodological considerations

Given the results of the three-level reconstruction (Fig. 4), it seems increasing the number of reconstructed levels requires an increasingly accurate delineation of the target region. While error propagation across multiple levels may lead to spurious results, at the same time, the definition of the seed region seems to play a key role in the robustness of the reconstruction. In both HCP and MASSIVE datasets, the seed regions were placed based on specific landmarks (brain stem and internal capsule). Incomplete segmentation of those regions would probably lead to reduced density of fiber pathways and, as a consequence, reduced quality of the reconstruction.

In this work, the analysis was fully focused on the application of the CST bundle given the well-defined anatomical landmarks that can be associated with the target and seed regions. Consequently, spurious pathways are often easy to detect which may not be the case for other bundles. For instance, to test the generalizability of MLFT, a reconstruction of the cingulum was performed with the MASSIVE dataset (Fig. S6). Despite the reconstruction being visually similar to the reference bundle from the ISMRM 2015 challenge, certain pathways may as well be spurious. For that reason, the MLFT reconstruction should be treated as a guidance, unless the target region is defined based on functional data. In any case, general prior knowledge of the anatomical configuration of the fiber bundle of interest is required to disambiguate interdigitating from branching pathways.

We would also like to stress that this approach in its current form is only suitable for bundle-specific applications or other cases that aim to investigate connections between two specific regions. As a consequence, it cannot be combined with algorithms for whole-brain reconstructions at the current moment.

### Future work

Although in this work we integrated the MLFT framework with the deterministic tractography, it can also be implemented for probabilistic tractography. In some probabilistic tractography methods such as iFOD2, for example, new directions are sampled at each propagation step from the fiber orientation distribution (concentrating around the peaks) only regarding peaks with an angular deviation lower than the pre-defined threshold (Fig. S5 in Online Resource). In this context, MLFT could be similarly applied to sample the propagation direction from the part of distribution outside the area conforming with the angular threshold when branching into the second level (Fig. S5 in Online Resource).

MLFT has shown promising results in healthy controls, but it remains unclear whether its performance will be maintained in presence of pathology especially with routinely acquired clinical data. Thus, evaluation of the algorithm in a clinical setting would also be beneficial.

It must be noted that in this work, we did not focus on devising an approach for estimating the required number of levels based on convergence criteria, which might be useful for clinical translation. For this study, those settings were identified empirically. Experiments were performed with up to 3 levels, however, there was not much change observed between results with 2 and 3 levels. One of the possible future directions of this work could be to introduce microstructural information in analogy to the dynamic seeding approach [58], which may allow for the automatic estimation of the number of levels, and which could facilitate the identification of valid branches.

## Supplementary Information

Below is the link to the electronic supplementary material.

Supplementary file1 (DOCX 14676 KB)
