# Supplementary material for: Anatomically informed multi-level fiber tractography for targeted virtual dissection
Source: MAGMA. 2022 Jul 29;36(1):79–93. doi: 10.1007/s10334-022-01033-3 (PMC9992235; doi:10.1007/s10334-022-01033-3)
Supplement: Supplementary file 1 — Supplementary file1 (DOCX 14676 KB) [file 10334_2022_1033_MOESM1_ESM.docx]

**Anatomically informed multi-level fiber tractography for targeted virtual dissection**

Short running title: Anatomically informed MLFT

Andrey Zhylka^1,*^, Alexander Leemans^2^, Josien P.W. Pluim^1^., Alberto De Luca^2,3^

^1^Biomedical Engineering, Eindhoven University of Technology, Eindhoven, The Netherlands

^2^Image Sciences Institute, University Medical Center Utrecht, Utrecht, The Netherlands

^3^Neurology Department, UMC Utrecht Brain Center, University Medical Center Utrecht, Utrecht, The Netherlands

*Corresponding author: Rondom 70, 5612AP, Eindhoven, The Netherlands, *e-mail:* [a.zhylka@tue.nl](mailto:a.zhylka@tue.nl)

Keywords: diffusion MRI, tractography, fiber orientation distribution, FOD, peak selection, brain white matter

Funding: Andrey Zhylka is supported by the European Union's Horizon 2020 research and innovation program [grant number 765148].


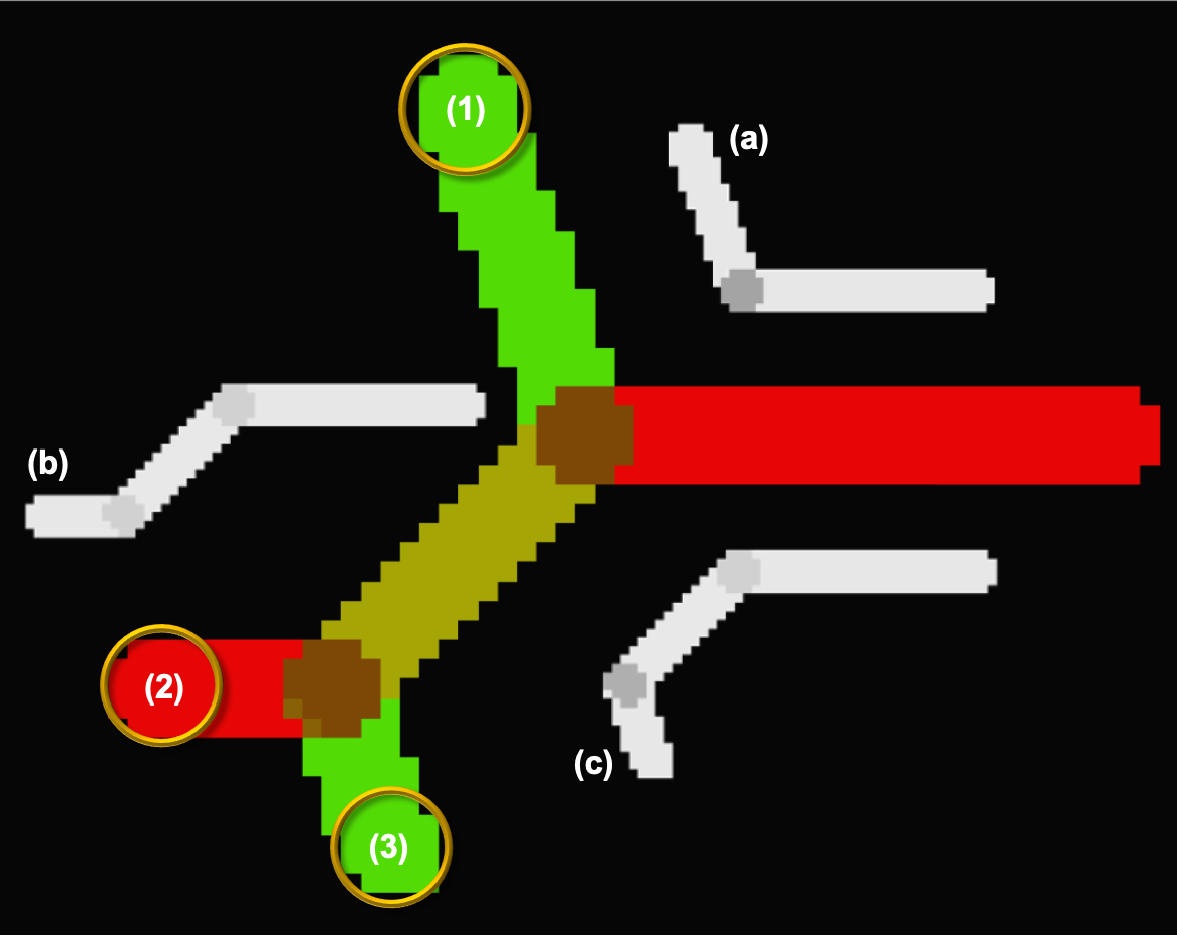


**Fig.** S1 A representation of the numeric phantom (colored based on diffusion direction and FA value) with two branching points. It consists of three individual fibers (a-c, colored according to FA value) with corresponding endpoint regions (1-3)


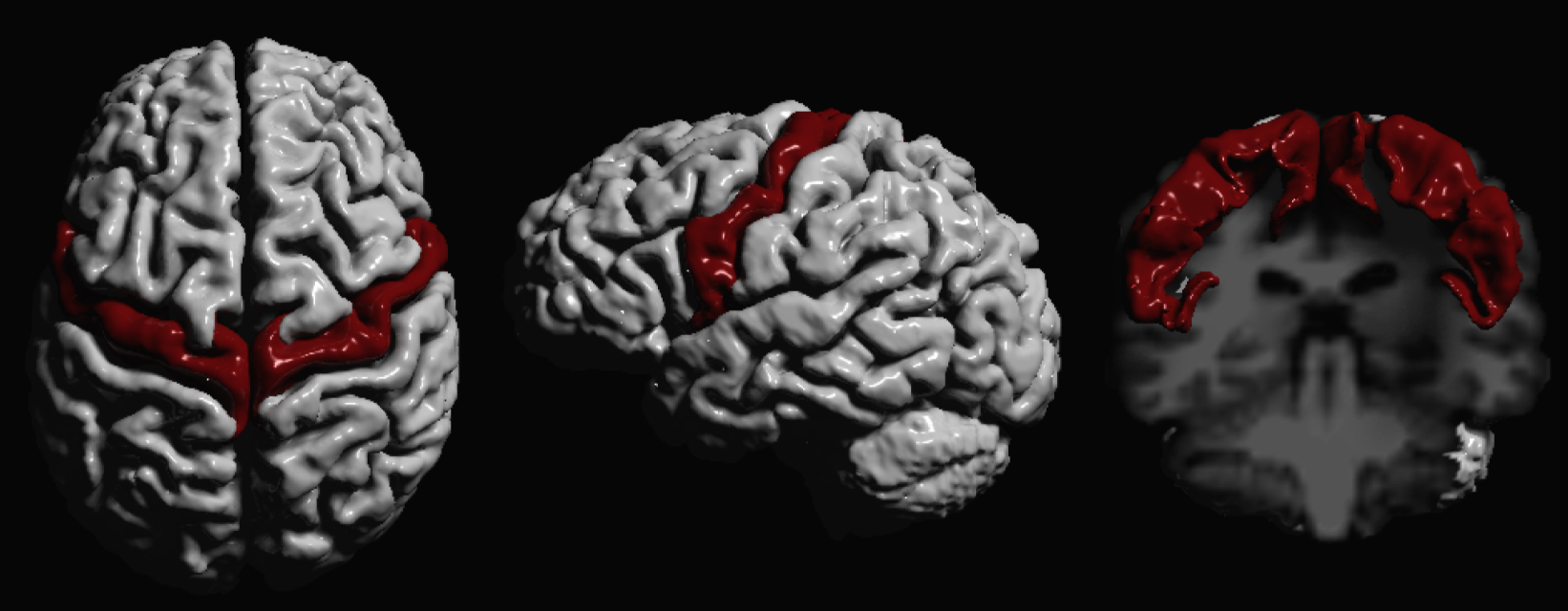


**Fig.** S2 The target cortical region. To reconstruct the corticospinal pathways, the motor cortex (red) was delineated for both hemispheres with FreeSurfer


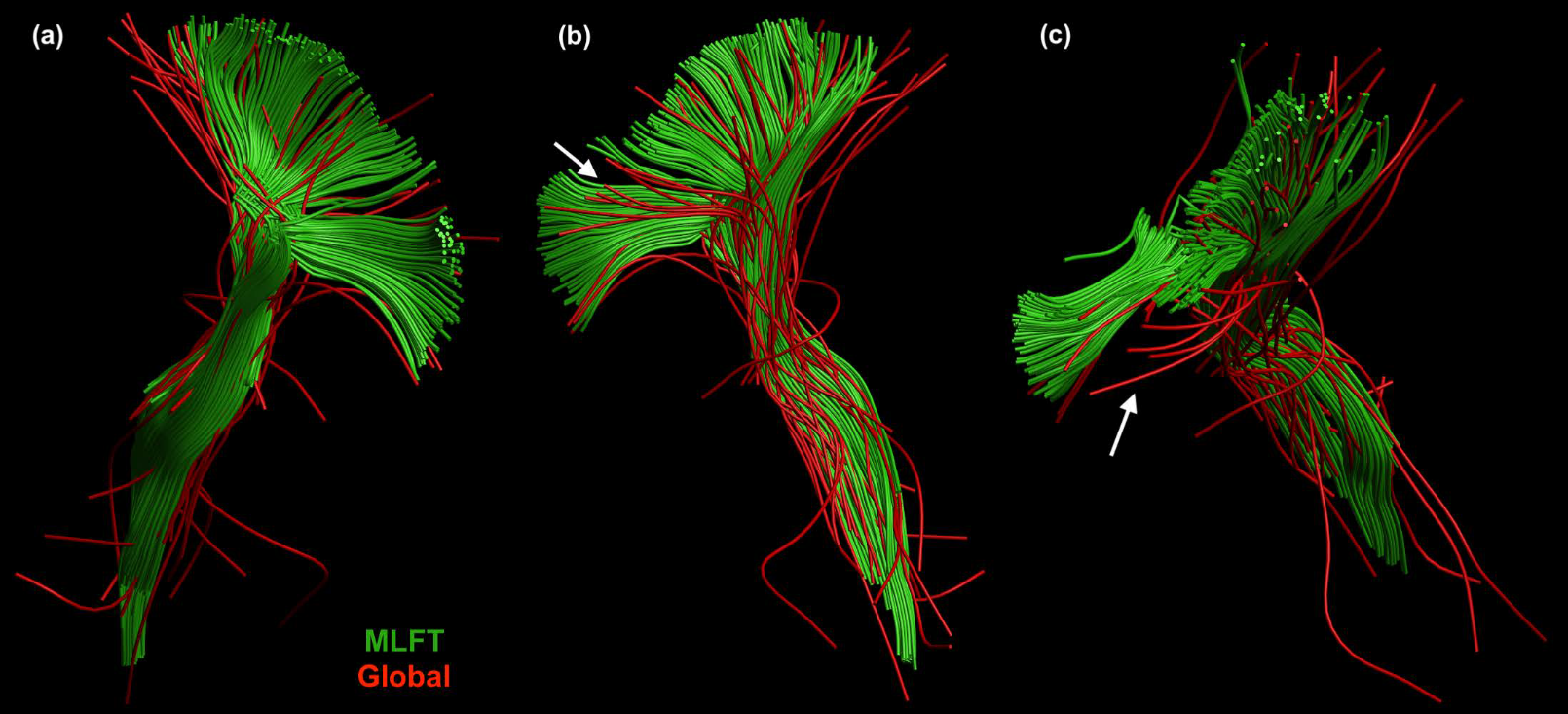


**Fig.** S3 Comparison of the left CST reconstructions obtained by MLFT (green) and GT (red) using the MASSIVE dataset. (a) The reconstruction by GT is sparser, but it provides additional coverage towards the approximate leg area unlike the MLFT reconstruction. (b) Pathways delineated by GT generally follow the same trajectory of the bundle reconstructed by MLFT but with smoother branching turns. (c) Some of the GT-produced pathways that are reaching the face motor area (white arrows in (b) and (c)) are shifted towards posterior part of the brain and are not completely aligned with others as well as with the corresponding part of the MLFT-reconstructed fanning


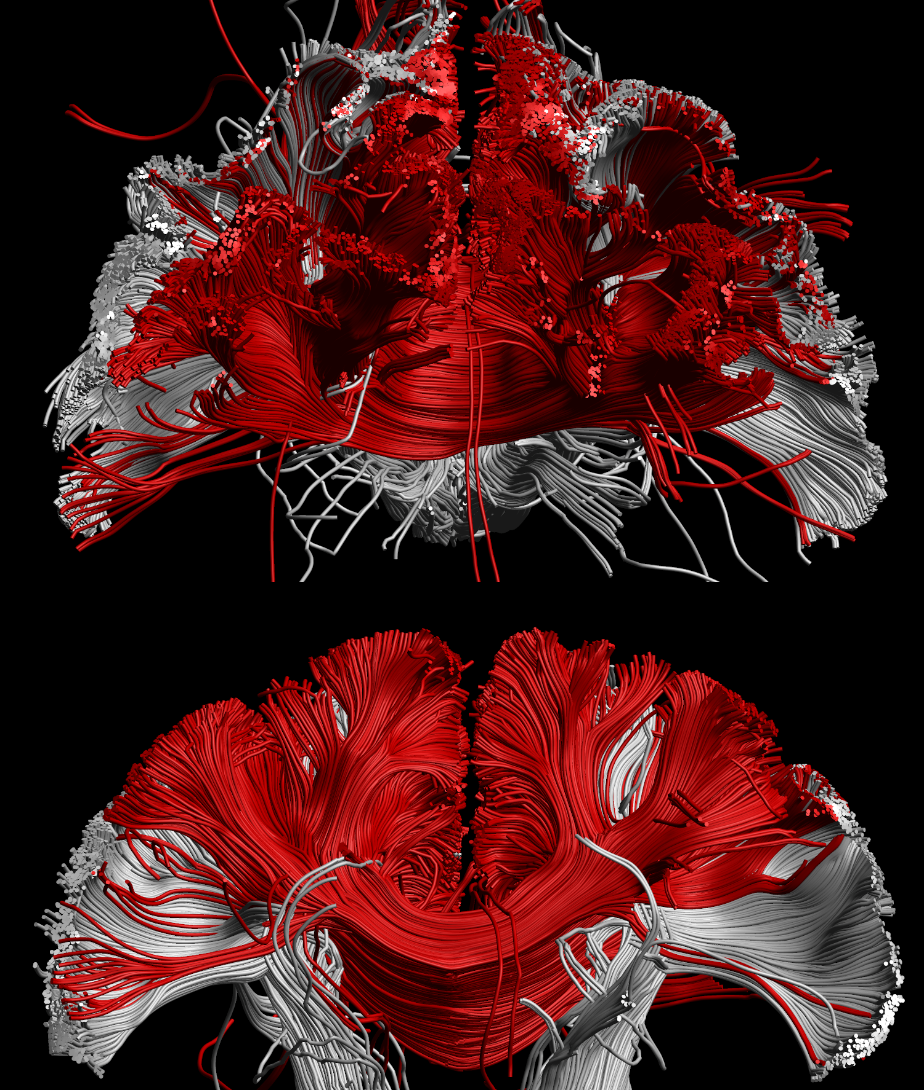


**Fig.** S4 The CST bundle (white) reconstructed by MLFT, and the CC bundle (red) reconstructed by whole-brain CSD-based tractography using a subject from the HCP dataset. The CST bundle was reconstructed using MLFT while the part of the CC bundle was extracted from whole-brain reconstruction obtained with the same parameters as in case of the MLFT reconstruction. An overlap can be seen in superior lateral part of motor cortex as well as occasionally in its temporal part. As it is known that the CC also originates from the motor cortex, the results support the ambiguity of whether the second-level branches the CST reconstruction belong to the CC or the CST, as they were not reconstructed by the deterministic CSD-based tractography in either cases given the same parameter settings


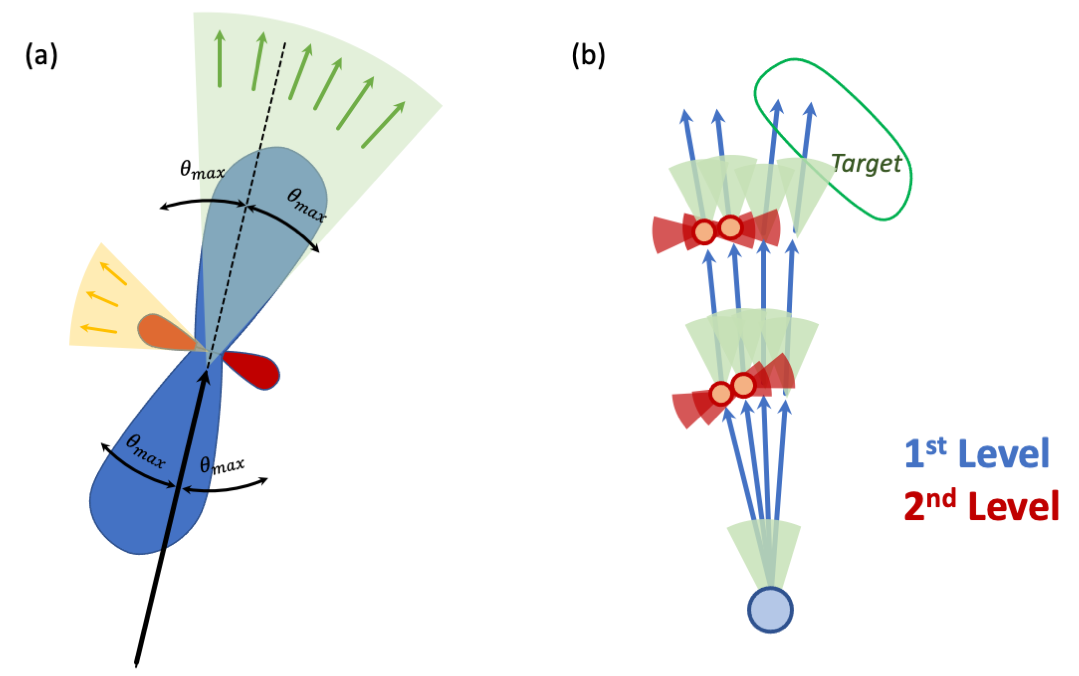


**Fig.** S5 (a) In probabilistic tractography new step directions are sampled into the directions with higher probability (green arrows) and then also constrained with an angular threshold ($\theta_{max}$). (b) Applying MLFT approach to probabilistic tractography at the second level would require sampling into the directions of the peak with deviation that is higher than the threshold (red area)

**Fig.** S6 The cingulum bundles reconstructed by the deterministic CSD-based ap- proach (left) and MLFT (right) from the same seed region (red) using the MASSIVE dataset. The cingulum bundle from the ISMRM 2015 challenge ground truth data [1] is shown in the bottom left corner for anatomical reference. The delineated pathways correspond better to the physical structure of the cingulum in comparison to the segmentation obtained by deterministic CSD-based tracking. The results are characterized by extensively improved fanning in the anterior and posterior parts of the bundle [2]. The seeds were located on the edge of Broadmann areas 23 and 24. The number of seeding points was selected empirically (5x5 grid subsampling at single slice level was used). The target region was chosen based on the White Matter Query Language [3] cingulum query description adjusted to include only the regions containing the end points of the bundle. Tracking parameters were set as in the Experiment 3 except for the FOD peak value threshold being set to 0.01. The target region (yellow, bottom right) included the following cortex parts: lateral orbitofrontal, lingual, inferior parietal and medial orbitofrontal cortex, parsopercularis, precuneus, superior frontal cortex, cuneus, entorhinal, fusiform, parahippocampal cortex.

## References

1. Maier-Hein KH, Neher P, Houde JC, et al (2017) The challenge of mapping the human connectome based on diffusion tractography. Nat Commun 8:1349
2. Catani M, de Schotten MT (2008) A diffusion tensor imaging tractography atlas for virtual in vivo dissections. Cortex 44:1105–1132
3. Wassermann D, Makris N, Rathi Y, et al (2016) The white matter query language: a novel approach for describing human white matter anatomy. Brain Struct Funct 221:4705–4721
